# Growth of *Staphylococcus epidermidis* on the Surface of Teatcups from Milking Parlours

**DOI:** 10.3390/microorganisms9040852

**Published:** 2021-04-15

**Authors:** Eleni I. Katsarou, Angeliki I. Katsafadou, Theodoros Karakasidis, Dimitris C. Chatzopoulos, Natalia G. C. Vasileiou, Daphne T. Lianou, Vasia S. Mavrogianni, Efthymia Petinaki, George C. Fthenakis

**Affiliations:** 1 Veterinary Faculty, University of Thessaly, 43100 Karditsa, Greece; elekatsarou@vet.uth.gr (E.I.K.); dlianou@vet.uth.gr (D.T.L.); vmavrog@vet.uth.gr (V.S.M.); 2 Faculty of Public and Integrated (One) Health, University of Thessaly, 43100 Karditsa, Greece; agkatsaf@vet.uth.gr (A.I.K.); vetdchatzop@gmail.com (D.C.C.); 3 Faculty of Physics, University of Thessaly, 35131 Lamia, Greece; thkarak@gmail.com; 4 Faculty of Animal Science, University of Thessaly, 41110 Larisa, Greece; vasileiounat@gmail.com; 5 University Hospital, University of Thessaly, 41110 Larisa, Greece; petinaki@med.uth.gr

**Keywords:** biofilm, cattle, mastitis, milking parlour, model, rubber, sheep, silicone, *Staphylococcus*, teatcup

## Abstract

The growth of two *Staphylococcus epidermidis* isolates (one biofilm-forming and one not) on teatcups for cattle (made of rubber) or sheep (made of silicone) were assessed in nine multiplicates for 24 h post-smearing on the teatcup surface. Staphylococci were smeared on an area of 0.0003142 m^2^ on the material and their growth and expansion further on were monitored for 24 h. There were no differences in the frequency of recoveries between the two isolates (*p* > 0.82 for all comparisons). There were more recoveries from sheep teatcups than from cattle teatcups: 1280/1728 (74.1%) versus 942/1728 (54.5%), for both isolates (*p* < 0.0001). Significance was observed only 6 h to 15 h after smearing (*p* < 0.0001 for all comparisons). The median speed of linear dissemination of the isolates was 0.00000021 m s^−1^ on cattle teatcups and 0.00000033 m s^−1^ on sheep teatcups (*p* < 0.0001). The increased growth and faster expansion of staphylococci on silicone teatcups raise important points from a clinical viewpoint. The model could be used in the testing of staphylococcal growth in the material of milking parlours in various conditions.

## 1. Introduction

Bacterial adhesion and growth on various surfaces are common in everyday life and may have significant consequences. For example, bacterial adhesion and growth on plastic medical devices can be a cause of bacterial infections in people [1,2]. Another example is the bacterial adhesion and growth on kitchen utensils, which have been incriminated in the contamination of food during preparation [3]. Nevertheless, the role and the involvement of the material on the adhesion and subsequent bacterial dissemination have not been fully clarified.

In a recently published paper [4], it was found that staphylococci were recovered from 23% of the teatcups in milking parlours in sheep and goat farms. The majority (87%) of the organisms recovered included biofilm-forming isolates. Biofilm formation by the staphylococcal isolates helped them to survive even during unfavourable conditions, e.g., treatment with antimicrobial chemicals. Indeed, the removal of bacterial biofilms by using standard cleaning and sanitation procedures has been found to be difficult or impossible [5].

The growth of bacterial cultures on teatcups and their spread thereon have not been thoroughly investigated. Based on current knowledge of staphylococcal biology, it is reasonable to postulate that staphylococci could remain on teatcups for some time depending on the daily frequency of milking sessions. The presence of skin lesions on the teats, which might have been caused during machine milking, has been found to predispose the animals to bacterial invasion into the mammary parenchyma and mastitis [6]. Staphylococci, which had survived on the teatcups, can invade into the teat during the subsequent milking session and then into the mammary parenchyma of animals, causing mastitis. The reverse pressure gradient is a mechanism by which this can occur; specifically, pressure differences that may occur for around 0.02 to 0.05 s in the milking system during the milking session can lead to an influx of bacteria present around the teat orifice (i.e., on the teatcups) into the teat canal [4,7].

The objective of the current work was to evaluate the differences in the growth of *Staphylococcus epidermidis*, a confirmed mammary pathogen for cattle and sheep, on teatcups used in milking parlours on farms with these animals.

## 2. Materials and Methods

### 2.1. Staphylococcal Isolates

Two *Staphylococcus epidermidis* isolates were used in the study. Both isolates had been recovered from milk samples from ewes with subclinical mastitis in Greece [8]. Subclinical mastitis was defined in ewes with no clinical mammary signs, in which a bacteriologically positive milk sample ((a) >10 colonies of the same organism and (b) no more than two different types of colonies) with concurrently increased leucocyte numbers therein (as detected by the California Mastitis Test (score ≥ l)) and increased neutrophil and lymphocyte proportions (≥65% of all leukocytes) [9]. Both isolates were recovered from flocks on which machine milking was applied.

Established bacteriological techniques were used for primary isolation; then, provisional identification of the isolates as *Staphylococcus* spp. was performed. The isolates were identified to the species level using the Vitek^®^ 2 automated system (BioMerieux, Marcy-l’-Étoile, France). Both isolates were found to be susceptible to antimicrobial agents (ampicillin, azithromycin, cefoxitin, clarithromycin, clindamycin, erythromycin, fosfomycin, fusidic acid, penicillin, tetracycline, and trimethoprim-sulfamethoxazole), after appropriate testing, which was performed by means of the Vitek^®^ 2 automated system. Moreover, both isolates were found to belong to sequence type (ST) 152 of the organisms, as established by means of multi-locus sequence typing (MLST) [10].

Both isolates were evaluated for in vitro biofilm-formation. Two techniques were employed. Initially, the isolates were cultured on Congo red agar and their colonial appearance was evaluated. Then, they were tested by means of the microplate adhesion method [11]. On Congo red agar plates, black colonies with dry crystalline consistency indicated a biofilm-forming isolate (isolate A), whilst colonies that remained pink, were considered to reflect a non-biofilm-forming isolate (isolate B) [12]. During the microplate adhesion method, the median ratios of the optical density of each test isolate to the optical density of the positive control isolate (*Staphylococcus aureus* ATCC25923) were 1.67 (isolate A) and 0.88 (isolate B). Based on the combination of results of the two techniques [11], one isolate was found to be biofilm-forming (A), whilst the other was not (B).

### 2.2. Test Material (Teatcups)

New teatcups were used as a test material. Teatcups for milking parlours for cattle and sheep were used; the former were made of rubber and the latter of silicone. Out of each teatcup, one square (8 cm × 8 cm) piece of material was cut.

**Figure 1:**
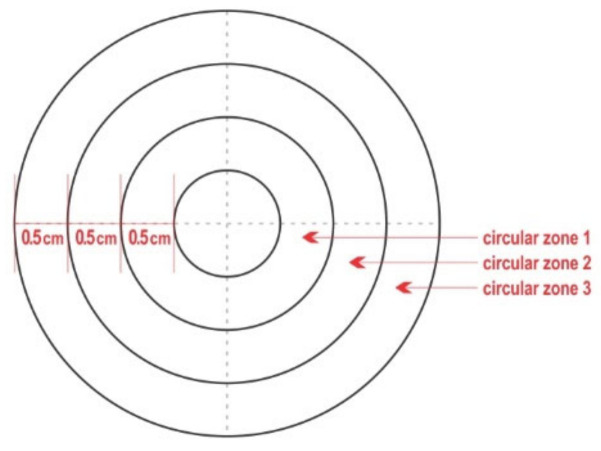
Diagram of the polar chart-type complex that was drawn on each piece of teatcup material.

A wooden board was used to pin thereon the pieces of teatcup material, which allowed them to remain stretched and facilitated all technical work. On the middle of each piece of teatcup material, a circle (⌀ = 1 cm) was drawn by using a pair of compasses. Then, another three concentric circles (⌀ = 2 cm, 3 cm, and 4 cm, respectively) were also drawn, i.e., the distance between each circle was 0.5 cm. A polar chart-type complex (hereafter termed ‘complex’) was formed, comprised of the inner circle and three outer circular zones (Figure 1). The circular zones were termed ‘circular zone 1′ for the innermost, ‘circular zone 2′ for the zone in the middle, and ‘circular zone 3′ for the outermost zone. Each complex of the four concentric circles was divided by drawing two diameters at a 90° angle between them; thus, four quadrants were created in each of the three outer zones, i.e., a total of 16 quadrants were created per chart.

Before inoculation of staphylococci on the material (smearing), the pieces were thoroughly cleaned with povidone-iodine (Betadine surgical scrub; Mundipharma AG, Basel, Switzerland), which after 10 min was rinsed with sterile phosphate-buffer-saline (PBS) pH 7.4 and fully dried with sterile gauzes.

After rinsing and immediately before smearing with staphylococci, the complex on the piece of teatcup material was swabbed throughout its surface with two sterile cotton swabs. These were then plated onto 5% sheep blood agar and *Staphylococcus* selective medium (mannitol agar). The media were incubated aerobically at 37 °C for 48 h; if there was no growth on the plates, they were re-incubated for another 24 h. If bacteria were recovered from any of the two pre-smearing swabs, experimental work carried out on that complex was discarded.

### 2.3. Experimental Work

Initially, each of the two *S. epidermidis* isolates was grown on Columbia blood agar; then, each isolate was inoculated into a vial with Tryptic Soy broth (BioMerieux) for aerobic incubation at 37 °C for 6 h. At that time, the contents of the vial were thoroughly mixed for 5 s in Vortex equipment (Velp Scientifica, Usmate, Italy), and thereafter, a sterile cotton swab was immersed in the broth, maintained therein for 3 s then withdrawn. Before taking the swab out of the vial, it was briefly pressed against the wall of the vial to drain excess fluid. The cotton swab was smeared on the centre of the complex on the piece of teatcup material to cover the entirety of the inner circle only. The bacterial content in the broth was subsequently calculated by means of the method of Miles and Misra [13] and found to vary between 4.22 × 10^9^ and 1.07 × 10^10^ c.f.u. mL^−1^ on all occasions.

After bacterial smearing, the piece of teatcup material was left at room temperature. This was maintained at 21 °C by means of an air-conditioning unit, functioning throughout this work.

After smearing, samples were collected every 3 h up to 24 h from each of the 12 quadrants in the three outer circular zones (4 quadrants in circular zone 1, 4 in circular zone 2, 4 in circular zone 3). Sampling involved lightly touching the surface of the material with a sterile cotton swab and immediately (within <1 s) withdrawing it from the material. On each occasion, sampling was performed twice in each quadrant with two different sterile swabs.

Each swab sample obtained from a quadrant of a circular zone was cultured in duplicate on 5% sheep blood agar and *Staphylococcus* selective medium (mannitol agar). The media were incubated aerobically at 37 °C for 48 h; if there was no growth on the plates, they were re-incubated for another 24 h. Bacterial isolation and initial identification were performed using standard methods [14,15]. Detection of at least one staphylococcal colony morphologically similar to the challenge isolates (*S. epidermidis*) from at least one of the two swab samples obtained on each sampling occasion was considered to indicate the presence of the organism on the piece of teatcup material on the quadrant of the complex from where the swab sample had been obtained.

For each isolate, nine (9) multiplicates were performed on teatcups for cattle and nine (9) multiplicates on teatcups for sheep. Further, control complexes, on which no bacteria were smeared, were also included in triplicate in each experiment. 

### 2.4. Data Management and Statistical Analysis

In the present study, there is difficulty with attempting to estimating the coverage of the quadrants of each circular zone. As there was an interval between sampling points, it is not possible to know what happened between the two sampling points. In addition, it was considered that isolation of staphylococci during swab sampling of a quadrant of a circular zone on the piece of teatcup material was not equivalent to full coverage of that quadrant with staphylococci. Therefore, the following definitions were initially made:

- ‘Isolation of staphylococci from the piece of teatcup material’ was equivalent to ‘presence of staphylococci on the piece of teatcup material’ on the respective quadrant that was sampled;
- A quadrant of a circular zone was considered to have been contaminated with staphylococci, if the bacteria were isolated from a swab sample;
- If no staphylococci were isolated on one sampling point from a quadrant and then isolated on the next sampling point, the contamination of the quadrant was deemed to have taken place halfway between the two sampling points;
- A quadrant of each circular zone was considered to have been fully covered with staphylococci if the bacteria were isolated from the respective concentric quadrant immediately outside it; for example, a quadrant in circular zone 1 was considered to have been fully covered if staphylococci were recovered from the respective quadrant in circular zone 2;
- In view of the design of the complex, it was possible to evaluate the full coverage of the two inner circular zones only (circular zone 1 and circular zone 2 and their combination);
- If staphylococci were not isolated on one sampling point from a quadrant and then isolated on the next sampling point, then the full coverage of the respective concentric quadrant immediately inside it was deemed to have taken place halfway between the two sampling points; for example, when staphylococci were recovered from a quadrant in circular zone 3, it was considered that the respective quadrant in circular zone 2 had been fully covered.

Based on the above, it was possible to calculate the speed of the linear dissemination of a staphylococcal isolate through a quadrant, at progressive 1.5 h time slots after smearing it on the material. Then, the average speed of the linear dissemination of an isolate through a complex was calculated as the average in the four quadrants (i.e., in the four directions).

With regard to the area of the complex, the area οf each of the three circular zones, from the innermost to the outermost, was 0.0009424 m^2^ (circular zone 1), 0.0015708 m^2^ (circular zone 2), and 0.0021990 m^2^ (circular zone 3), respectively. Hence, the area of each quadrant of these zones was 0.0002356 m^2^ for circular zone 1 and 0.0003927 m^2^ for circular zone 2.

Based on the above, it was possible to calculate an estimate of the length of time necessary for the full coverage of each quadrant in circular zone 1 and circular zone 2. It was also possible to calculate the surface of the complex that had been covered at various time-points post-smearing.

Data were entered into Microsoft Excel and analyzed using SPSS v. 21 (IBM Analytics, Armonk, NY, USA). Basic descriptive analysis was performed. Frequencies were evaluated in tables of cross-categorised data by use of Pearson chi-square test or Fisher exact test, as appropriate. Results of the speed of linear dissemination and surface covered were compared between isolates, material, or circular zones by using the Kruskal–Wallis test. Statistical significance was defined at *p* < 0.05.

## 3. Results

No bacteria were recovered from any of the swab samples obtained after rinsing the material with PBS and before smearing the staphylococcal isolates thereon.

After smearing, staphylococci were consistently recovered from the swab samples obtained from the inner circle of the complex on all sampling occasions, i.e., from 3 h to 24 h post-inoculation. This was consistent for all nine multiplicates performed, for both isolates and for the teatcups for cattle and sheep. In contrast, no staphylococci were recovered from any complex that had not been smeared with staphylococci at any sampling point (*p* < 0.0001 for all comparisons versus complexes on which staphylococci were smeared).

There were no differences in the frequencies of staphylococcal recoveries between the two isolates, neither on any specific sampling occasion, nor cumulatively. Cumulative recoveries were 1110/1728 (64.2%) for isolate A (472/864 = 54.7% from cattle teatcups and 638/864 = 73.8% from sheep teatcups) and 1112/1728 (64.4%) for isolate B (470/864 = 54.4% and 642/864 = 74.3%, respectively) (*p* > 0.82 for all comparisons).

**Figure 2:**
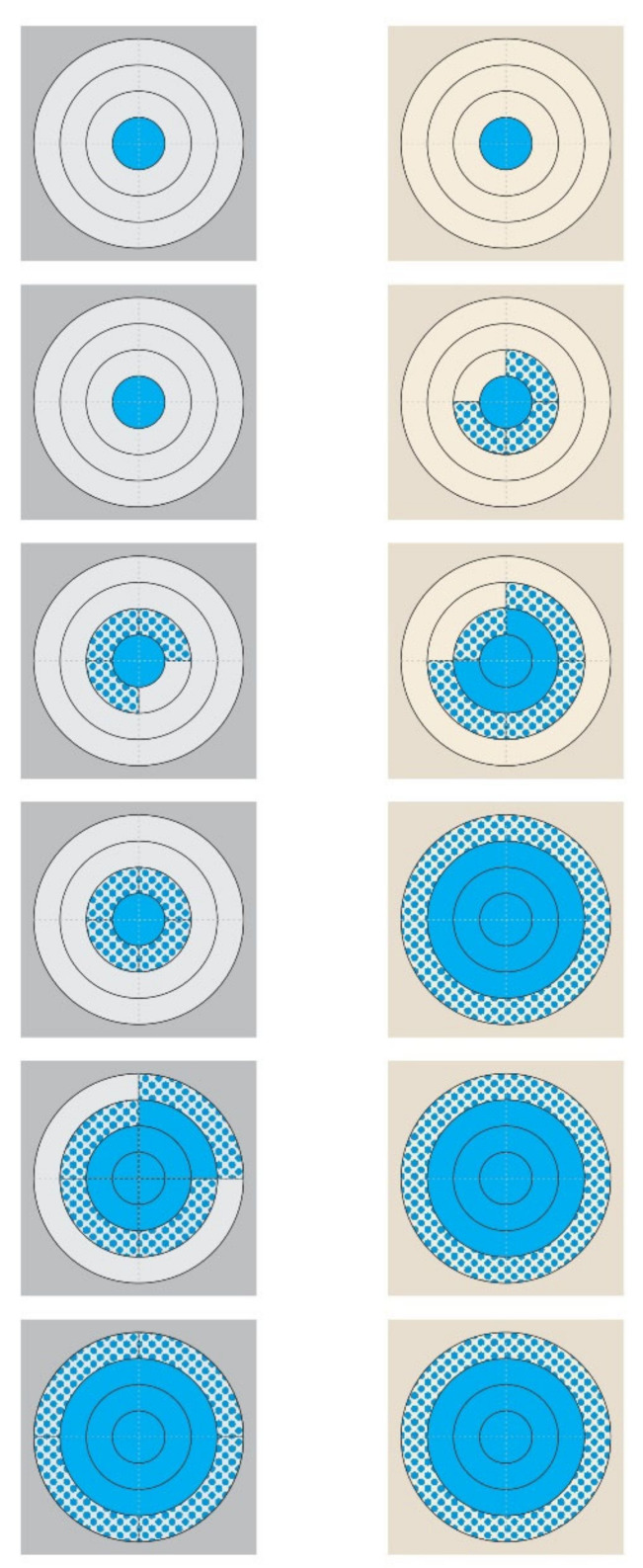
Diagrammatic presentation of the progressive growth of *S. epidermidis* from two polar chart-type complexes on pieces of teatcup material. (Legend: from top to bottom: 3 h, 6 h, 9 h, 12 h, 15 h, and 18 h post-smearing; (**left**): grey-coloured complex, cattle teatcup; (**right**): cream-coloured complex, sheep teatcup; blue motif pattern: the presence of *S. epidermidis* on a quadrant, blue full pattern: the coverage of a quadrant with *S. epidermidis.*)

There was evidence of increased staphylococcal recoveries from the teatcups for sheep compared to teatcups for cattle: 1280/1728 (74.1%) versus 942/1728 (54.5%) (*p* < 0.0001). This was consistent for both isolates: 638/864 (73.8%) versus 472/864 (54.6%) for isolate A and 642/864 (74.3%) versus 470/864 (54.4%) for isolate B (*p* < 0.0001 for all comparisons). With regard to sampling occasion, a significant difference was observed 6 h to 15 h after smearing (*p* < 0.0001 for all comparisons), whilst in samplings 3 h, 18 h, 21 h, and 24 h after smearing, no such difference was evident (Table 1, Figure 2, Table S1).

**Figure 3:**
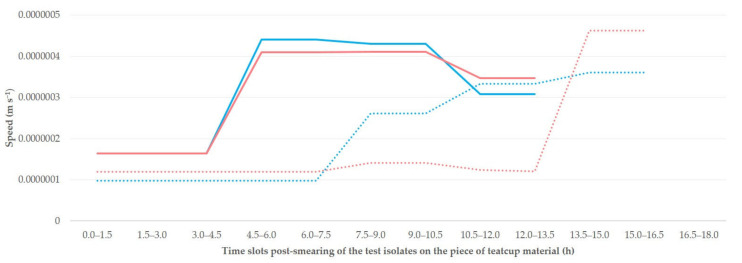
Median speed of linear dissemination of two isolates of *S. epidermidis* (isolate A: blue colour; isolate B: red colour; *p* > 0.25 for all comparisons) at progressive time slots after smearing of the isolates on pieces of teatcup material from milking parlours for cattle (dotted line) or sheep (solid line) (*p* < 0.003 for all comparisons).

The median speed of linear dissemination of the isolates throughout the study was 0.00000021 m s^−1^ on teatcups for cattle and 0.00000033 m s^−1^ on teatcups for sheep, for both isolates (*p* > 0.25 for all comparisons between isolates, *p* < 0.003 for all comparisons between teatcups for cattle and teatcups for sheep). The progressive changes in the speed of linear dissemination of the isolates (Figure 3) were also significant (*p* < 0.035 for all comparisons).

**Figure 4:**
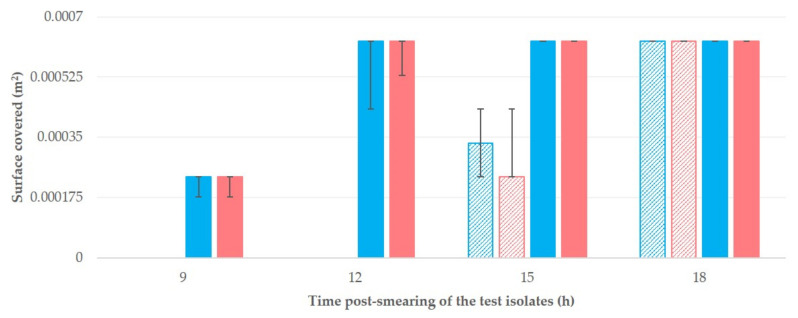
Median (min–max) surface covered (m^2^) by two isolates of *S. epidermidis* (isolate A: blue colour; isolate B: red colour; *p* > 0.35 for all comparisons) at different time-points after smearing on teatcups from milking parlours for cattle (massif pattern) or sheep (full pattern) (*p* < 0.01 for comparisons at 9 h, 12 h and 15 h post-smearing).

The median values of the surface covered for each isolate (A or B) and material (cattle teatcups or sheep teatcups) at various time-points are in Table 2 and Figure 4. The differences between isolates A and B were not significant (*p* > 0.35 for all comparisons). In contrast, the differences between teatcups for cattle and teatcups for sheep were significant (*p* < 0.0001 for comparisons at 9 h, 12 h, and 15 h post-smearing).

Finally, there were no differences in the frequencies of staphylococcal recoveries among the nine multiplicates during the evaluation of the same staphylococcal isolate assayed on the same material (*p* > 0.98).

## 4. Discussion

Bacterial growth and motility on surfaces is important for the survival of the microorganisms. Among these, *S. epidermidis* is considered to move by ‘darting’, which has been defined as a type of surface translocation produced by the expansive forces developed in an aggregate of cells inside a common capsule and resulting in the ejection of cells from the aggregate [16,17]. During darting, the bacteria cluster on a surface and grow. When growth overwhelms the adhesive forces keeping the bacteria within the cluster, some bacterial cells are ejected and new colonies are formed, resulting in expansion of the surface occupied by the bacteria [16,17]. This is a dissemination means unique to *S. epidermidis*, although it has been indicated that it might occur also in *S. xylosus* [18].

Various factors can affect staphylococcal growth and dissemination on surfaces. Bacteria can adhere to the surface through physicochemical interactions [19], which include cell surface hydrophobicity [20,21] and charge [22]. For example, for *S. aureus*, it has been found that the bacteria float on the water present on a surface and can spread rapidly [23]. In this model, future studies can include the use of milk or organic residues on the teatcups, which would simulate conditions occurring in milking parlours after improper cleaning procedures.

The present results have indicated that biofilm formation by one of the test isolates possibly does not offer any advantage in the dissemination of the bacteria on the test surfaces. Biofilm formation is a process by means of which microorganisms attach onto a surface and produce extracellular polymers that facilitate attachment and matrix formation [24]. In this case, two isolates in the two extremes, i.e., a non-biofilm-forming and a strong biofilm-forming isolate, were included, whilst all other properties of the two isolates were similar: same ST (ST152), full susceptibility to antimicrobial agents, and recovery in farms applying machine milking. This lack of effect is compatible with the mode of darting, during which dissemination takes place by ejection rather than attachment to the surface. Biofilm formation plays a role in staphylococcal survival during cleaning, as corroborated by the findings of our previous study in which most (87%) staphylococcal isolates recovered from teatcups were biofilm-forming [4], but not for the subsequent dissemination on the teatcups.

The results provide evidence that the dissemination of staphylococci on the teatcups differed during the immediately post-contamination stage according to the material of the teatcups. Rubber biodegradation is a slow process and utilization of rubber by bacteria is not effective and leads to slow growth [25]. Moreover, rubber has a low water content, which would further limit staphylococcal growth [26,27]. In practice, this can have consequences in clinical circumstances; if teatcups were properly cleaned and dried post-milking, there would be little bacterial dissemination.

The increased dissemination of staphylococci on silicone teatcups is important from a clinical viewpoint. In previous relevant studies, silicone surfaces were found to promote better dissemination of staphylococci compared to acrylic surfaces [28]. Moreover, Kodjikian et al. [29] have reported that, among various biomaterials used for ophthalmic lenses, silicone was the one found with the heaviest bacterial populations after intraocular implantation. Staphylococcal growth was also reported by Kim et al. [30] to have occurred in silicone tubes removed from dacryocystorhinostomy patients.

Staphylococci present on teatcups pose a significant risk for intramammary infection and the development of mastitis. The bacteria attach to the skin of the teats during milking, whence they may invade the teat. Moreover, pressure differences (1.5 to 7.0 kPa) occurring in the system during the milking procedure, even for very short periods (0.02 to 0.05 s) can lead to an ‘aspiration’ of staphylococci around the teat orifice and on the teatcups [7]. It is possible that the increased dissemination of staphylococci on teatcups for sheep, in comparison to teatcups for cattle, may, to some extent, contribute to the higher frequency of staphylococcal mastitis in ewes (over 75% of all cases of mastitis) [8,31] in comparison to cows, in which animals streptococci and environmental pathogens (e.g., *Escherichia coli*) are also frequent causal agents of infection [32,33].

The approach that was employed in the current study indicates that this model may be used for the evaluation of bacterial growth on teatcups in various conditions and circumstances. For example, environmental conditions could be tested by exposing the material to a range of temperatures and relative humidity, in order to evaluate varying ambient conditions, whilst chemicals could also be tested for their ability to clear or limit bacterial growth on the teatcups.

New teatcup material were employed and tested. The idea was to study the growth of the bacteria on unused teatcups and establish baseline findings. Further studies would need to include used teatcups, at various stages of tearing. Fissures present on the surface of used teatcups, which increase the total area of the material may, possibly, affect the growth of the bacteria. The evaluation of the growth of staphylococci on used teatcups will result in formulating recommendations regarding the usage time for teatcups in accord with their condition.

## 5. Conclusions

The results have provided clear evidence that isolates of *S. epidermidis* have increased growth and dissemination on teatcups for sheep (silicone) than on teatcups for cattle (rubber). The findings underline the importance of correct post-milking cleaning of the milking parlour in order to fully eliminate any staphylococcal foci thereon. Moreover, the use of detergent is important, as it would completely clean any traces of organic material (e.g., milk), which could serve to support the growth of the surviving bacteria. The model could be used in the testing of staphylococcal growth in the material of milking parlours. Future studies can evaluate the significance of various parameters, e.g., differences between bacterial isolates, presence of water or milk on the teatcup material, environmental conditions (temperature, humidity), thus simulating many of the field conditions and presenting information regarding the growth of the bacteria in various conditions.

## Figures and Tables

**Table 1:** Summary of recoveries of two *S. epidermidis* isolates after smearing on teatcups for cattle or sheep.

Time after Smearing	Total Recoveries	
	Isolate A	Isolate B
Teatcups for cattle		
3 h	0/108	0/108
6 h	0/108	0/108
9 h	32/108	33/108
12 h	36/108	36/108
15 h	80/108	77/108
18 h	108/108	108/108
21 h	108/108	108/108
24 h	108/108	108/108
Total	472/864	470/864
Teatcups for sheep		
3 h	0/108	0/108
6 h	33/108	33/108
9 h	71/108	71/108
12 h	102/108	106/108
15 h	108/108	108/108
18 h	108/108	108/108
21 h	108/108	108/108
24 h	108/108	108/108
Total	638/864	642/864
Grand total	1110/1728	1112/1728

**Table 2:** Median (min–max) surface covered (m^2^) by two isolates of *S. epidermidis* at different time-points 9 h–18 h) after smearing on teatcups from milking parlours.

Time after Smearing	Isolate A	Isolate B
Teatcups for cattle		
9 h	0.000000 ^a^ (0.000000–0.000000)	0.00000 ^a^ (0.00000–0.00000)
12 h	0.000000 ^b^ (0.00000–0.000000)	0.00000 ^b^ (0.00000–0.00000)
15 h	0.000334 ^c^ (0.000236–0.000432)	0.000236 ^c^ (0.000236–0.000432)
18 h	0.000628 (0.000628–0.000628)	0.000628 (0.000628–0.000628)
Teatcups for sheep		
9 h	0.000236 ^a^ (0.000177–0.000236)	0.000236 ^a^ (0.000177–0.000432)
12 h	0.000628 ^b^ (0.000628–0.000628)	0.000628 ^b^ (0.000530–0.000628)
15 h	0.000628 ^c^ (0.000628–0.000628)	0.000628 ^c^ (0.000628–0.000628)
18 h	0.000628 (0.000628–0.000628)	0.000628 (0.000628–0.000628)

^a–c^ values within the same column marked with the same superscript are significantly different between them with *p* < 0.01.

## Data Availability

All data associated with this manuscript are given in the manuscript or in the Supplementary Materials.

## Supplementary Materials

The following are available online at https://www.mdpi.com/article/10.3390/microorganisms9040852/s1, Table S1: Detailed results of recoveries of two *S. epidermidis* isolates from teatcups for cattle or sheep.
