# Supplementary material for: Growth of Staphylococcus epidermidis on the Surface of Teatcups from Milking Parlours
Source: Microorganisms. 2021 Apr 15;9(4):852. doi: 10.3390/microorganisms9040852 (PMC8071573; doi:10.3390/microorganisms9040852)
Supplement: Supplementary file 1 [file microorganisms-09-00852-s001.zip › microorganisms-1188781-SI.pdf]

Communication

Growth of *Staphylococcus epidermidis* on the Surface of Teat-cups from Milking Parlours

Eleni I. Katsarou, Angeliki I. Katsafadou, Theodoros Karakasidis, Dimitris C. Chatzopoulos, Natalia G.C. Vasile-iou, Charalambia K. Michael, Vasia S. Mavrogianni, Efthymia Petinaki and George C. Fthenakis

Table S1. Detailed results of recoveries of two *S. epidermidis* isolates from teatcups for cattle or sheep.

Time after smearing	Recoveries from circular zone 1	Recoveries from circular zone 2	Recoveries from circular zone 3	Total recoveries
		**Teatcups for cattle**		
		Isolate A		
3 h	0/36	0/36	0/36	0/108
6 h	0/36	0/36	0/36	0/108
9 h	32/36	0/36	0/36	32/108
12 h	36/36	0/36	0/36	36/108
15 h	36/36	36/36	8/36	80/108
18 h	36/36	36/36	36/36	108/108
21 h	36/36	36/36	36/36	108/108
24 h	36/36	36/36	36/36	108/108
Total	212/288	144/288	116/288	472/864
		Isolate B		
3 h	0/36	0/36	0/36	0/108
6 h	0/36	0/36	0/36	0/108
9 h	33/36	0/36	0/36	33/108
12 h	36/36	0/36	0/36	36/108
15 h	36/36	36/36	5/36	77/108
18 h	36/36	36/36	36/36	108/108
21 h	36/36	36/36	36/36	108/108
24 h	36/36	36/36	36/36	108/108
Total	213/288	144/288	113/288	470/864
		**Teatcups for sheep**		
		Isolate A		
3 h	0/36	0/36	0/36	0/108
6 h	33/36	0/36	0/36	33/108
9 h	36/36	35/36	0/36	71/108
12 h	36/36	36/36	30/36	102/108
15 h	36/36	36/36	36/36	108/108
18 h	36/36	36/36	36/36	108/108
21 h	36/36	36/36	36/36	108/108
24 h	36/36	36/36	36/36	108/108
Total	249/288	215/28	174/288	638/864
		Isolate B		
3 h	0/36	0/36	0/36	0/108

6 h	33/36	0/36	0/36	33/108
9 h	36/36	33/36	2/36	71/108
12 h	36/36	36/36	34/36	106/108
15 h	36/36	36/36	36/36	108/108
18 h	36/36	36/36	36/36	108/108
21 h	36/36	36/36	36/36	108/108
24 h	36/36	36/36	36/36	108/108
Total	249/288	213/288	180/288	642/864
